# *Eremogone* (Caryophyllaceae): new combinations for Old World species

**DOI:** 10.3897/phytokeys.50.4736

**Published:** 2015-06-02

**Authors:** Richard K. Rabeler, Warren L. Wagner

**Affiliations:** 1 University of Michigan Herbarium-EEB, 3600 Varsity Drive, Ann Arbor, MI 48108-2228, USA; 2 Department of Botany, MRC-166, National Museum of Natural History, Smithsonian Institution, P.O. Box 37012, Washington, DC 20013-9599, USA

**Keywords:** *Eremogone*, *Arenaria*, Caryophyllaceae

## Abstract

Twenty-one new combinations in *Eremogone* (Eremogoneae, Caryophyllaceae) are proposed to accommodate placement of all Old World taxa of Arenaria subg. Eremogone and *Eremogoneastrum* within *Eremogone*.

## Introduction

In their study of relationships in *Arenaria* L. and major lineages within the Alsinoideae, Harbaugh et al. (2010) found that members of Arenaria subg. Eremogoneastrum F.N. Williams and *Eremogone* (Fenzl) Fenzl, as well as Minuartia L. subg. Spergella (Fenzl) McNeill and *Thylacospermum* Fenzl, clustered in a clade that was both distantly related to the rest of *Arenaria* and sister to the Caryophylloideae. The tribe Eremogoneae was described in that paper to place this clade within the context of their tribal classification of the family.

Three subsequent molecular studies have confirmed the placement of the *Arenaria* and *Minuartia* species in Eremogoneae (Greenberg and Donoghue 2011; Dillenberger and Kadereit 2014; Sadeghian et al. in press). The first two studies included a similar complement of taxa in the tribe with the exception of *Thylacospermum*, which clustered with *Spergula arvensis* L. in those studies. While Harbaugh et al. (2010) proposed that the genus *Phlebanthia* Rchb. be resurrected for the species of Minuartia subg. Spergella, Dillenberger and Kadereit (2014) instead proposed new combinations in *Eremogone* for these taxa, noting there was not a clear segregation among the sampled taxa. We follow the results of Greenberg and Donoghue (2011) and Dillenberger and Kadereit (2014) by excluding *Thylacospermum* from *Eremogone* at this time.

*Eremogone*, as now defined, consists of the former Arenaria subg. Eremogone (~ 70 sp.), subg. Eremogoneastrum (22 sp.), and Minuartia subg. Spergella (3 sp.). Sadeghian et al. (in press) included a larger sample of taxa now placed in *Eremogone* than earlier studies; they concluded that infrageneric relationships were still unclear. While many combinations in *Eremogone* have been published (esp., Ikonnikov (1973, 1990) and Hartman and Rabeler (2004)), most members of Arenaria subg. Eremogoneastrum lack combinations in *Eremogone*. With active flora projects in India (Flora of India Checklist, in prep.) and China (Flora of China, e.g. Wu et al. 2001) bringing more information to light about these regions we feel that it is time to supply the 21 additional combinations to make all currently recognized taxa available in *Eremogone*.

The information about type specimens of the basionyms of the new combinations that we have included is based on examining protologues and searching major indices (Tropicos – http://www.tropicos.org/; JSTOR Global Plants – https://plants.jstor.org/) for extant specimens. Herbarium abbreviations follow Index Herbariorum (Thiers 2015). In cases where specimen deposition is not clearly stated in a protologue, we have added “?” after the abbreviation where, based on information about the location of the herbarium where the author worked and/or deposited their herbaria (see Index of Botanists – http://kiki.huh.harvard.edu/databases/botanist_index.html), we expect, but cannot confirm, a type specimen should be deposited.

In the cases where syntypes are cited, we have refrained from designating lectotypes. It is not a requirement for the names to be validly and effectively published and we consider those decisions should be made during the course of a taxon-level revision where a serious study of all specimens would lead to the best selections.

## Taxonomic part

### Eremogone

Taxon classificationPlantaeCaryophyllalesCaryophyllaceae

Fenzl, Vers. Darstell. Alsin. 13. 1833

#### Lectotype.

(see McNeill in Notes Roy. Bot. Gard. Edinburgh 24: 120. 20 Sep 1962): *Eremogone graminifolia* Fenzl, Vers. Darstell. Alsin. 37. 1833.

#### Description.

Plants perennial, sometimes densely cespitose or pulvinate or with a stout woody caudex, very rarely annual. Leaves filiform to subulate, often long-linear and grass-like, sometimes short and setaceous or needle-like, congested in vegetative rosettes and at or near base of flowering stems, margin sometimes scarious, apex often apiculate. Inflorescence of one or more terminal cymes, sometimes compressed to head-like, sometimes flowers solitary or paired. Flowers weakly perigynous. Sepals often hardened at base, veins 1–3, margins often white-scarious. Petals white, rarely pink. Floral glands (nectaries) at base of the antisepalous filaments often lobed, conspicuous.

### New combinations

#### Eremogone aksayqingensis

Taxon classificationPlantaeCaryophyllalesCaryophyllaceae

(L.H.Zhou) Rabeler & W.L.Wagner comb. nov.

urn:lsid:ipni.org:names:77147449-1

Arenaria aksayqingensis L.H.Zhou, Acta Biol. Plateau Sin. 6: 25. 1987.

##### Type.

CHINA. Xinjiang: Aksayqing, ca. 4900 m. Northwest Plat. Inst. Biol. Acad. Sin. Exped. 3723 (holotype, NWBI).

#### Eremogone commagenae

Taxon classificationPlantaeCaryophyllalesCaryophyllaceae

(Çeleb. & Favarger) Rabeler & W.L.Wagner comb. nov.

urn:lsid:ipni.org:names:77147450-1

Arenaria commagenae Çeleb. & Favarger, Candollea 44: 329. 1989.

##### Type.

TURKEY: Adiyaman, Mt. Nemrut Dagi, 1950 m, 5 July 1983, T. Ҫelebioğlu 83-8/1 (holotype, ISTF).

#### Eremogone depauperata

Taxon classificationPlantaeCaryophyllalesCaryophyllaceae

(Edgew.) Rabeler & W.L.Wagner comb. nov.

urn:lsid:ipni.org:names:77147452-1

Arenaria depauperata (Edgew.) H.Hara, J. Jap. Bot. 51: 129. 1976, non Gay (1846). *Stellaria depauperata* Edgew., Fl. Brit. India [J.D. Hooker] 1(2): 234. 1874.

##### Type.

INDIA: Alpine Sikkim Himalaya, Yeumtong in gravelly places, alt. 15000 ft.; J.D. Hooker s.n. (holotype, K?; possible isotype, GH, GH00353889 [JSTOR image!]).

#### Eremogone ferruginea

Taxon classificationPlantaeCaryophyllalesCaryophyllaceae

(Duthie ex Williams) Rabeler & W.L.Wagner comb. nov.

urn:lsid:ipni.org:names:77147453-1

Arenaria ferruginea Duthie ex Williams, J. Linn. Soc., Bot. 33: 410. 1898.

##### Type.

INDIA: Kumaon, Kali Valley, on rocks near Byans, 2800–3000 m, J.F. Duthie 2762 (BM?).

#### Eremogone festucoides

Taxon classificationPlantaeCaryophyllalesCaryophyllaceae

(Benth.) Rabeler & W.L.Wagner comb. nov.

urn:lsid:ipni.org:names:77147454-1

Arenaria festucoides Benth., Ill. Bot. Himal. Mts. 1: 81, pl. 21, f. 3. 1834.

##### Type.

Kunawar (K?).

#### Eremogone festucoides (Benth.) Rabeler & W. L. Wagner var. imbricata

Taxon classificationPlantaeCaryophyllalesCaryophyllaceae

(Bieb.) Rabeler & W.L.Wagner comb. nov.

urn:lsid:ipni.org:names:77147455-1

Arenaria festucoides Benth. var. imbricata (Bieb.) Edgew. & J.D. Hooker, Fl. Brit. India [J.D. Hooker] 1(2): 234. 1874. *Arenaria imbricata* M.Bieb., Fl. Taur.-Caucas. 1: 344. 1808.

##### Type.

Caucasus. circa Kobi (LE?).

#### Eremogone gerzensis

Taxon classificationPlantaeCaryophyllalesCaryophyllaceae

(L.H.Zhou) Rabeler & W.L.Wagner comb. nov.

urn:lsid:ipni.org:names:77147456-1

Arenaria gerzensis L.H.Zhou, Rep. Invest. Fl. Fauna Ah Li Reg. Tibet. 126. 1979.

##### Type.

CHINA. Xizang: Ngari Diqu, Geze, 4500–4700 m, collector unknown 4346 (holotype, NWBI).

#### Eremogone grueningiana

Taxon classificationPlantaeCaryophyllalesCaryophyllaceae

(Pax & K. Hoffmann) Rabeler & W.L.Wagner comb. nov.

urn:lsid:ipni.org:names:77147457-1

Arenaria grueningiana Pax & K. Hoffmann in Pax, Repert. Spec. Nov. Regni Veg. Beih. 12: 366. 1922.

##### Syntypes.

CHINA: Tschili (Hebei). Hsiau Wu tai schan, Felsen des Gipfels, 3050 m, 3 Aug 1912, H.W. Limpricht 600 (WRSL?, WU, WU0029891 [JSTOR image!]); Gipfelfelsen des Pe tai, 4050 m, Kalk, 26 Jun 1915, H.W. Limpricht 2563 (WRSL?, WU, WU0029892 [JSTOR image!]); Felsen des His tai, 3000 m, H.W. Limpricht 2982 (WRSL?). West-Tibet: Hor Tschango, Schtiala, Geröll des Schao kirr bu, 4700 m, H.W. Limpricht 2095 (WRSL?); Kanse, Tsokoma-Stock, Geröllhalden, 5000 m, H.W. Limpricht 2127 (WRSL?). Batang-Litang Pungtschamu, Felsen des Passes Dshagala, 5260 m, H.W. Limpricht 2262 (WRSL?).

This combination is proposed based on the placement of this taxon in subg. *Eremogone* in Wu et al. (2001). Pax and Hoffmann (Pax 1922) originally thought it was close to *Arenaria przewalskii* Maxim., placed in subg. *Dolophragma* (Fenzl) McNeill in Wu et al. (2001). Neither taxon has been included in a molecular analysis as of this writing.

#### Eremogone haitzeshanensis

Taxon classificationPlantaeCaryophyllalesCaryophyllaceae

(Tsui ex L.H.Zhou) Rabeler & W.L.Wagner comb. nov.

urn:lsid:ipni.org:names:77147458-1

Arenaria haitzeshanensis Tsui ex L.H.Zhou, Acta Biol. Plateau Sin. 13: 1. 1997.

##### Type.

CHINA. Sichuan: Dege, Haizi Shan, 3800 m., Sichuan Exped. D-7475 (holotype, WUG).

#### Eremogone ischnophylla

Taxon classificationPlantaeCaryophyllalesCaryophyllaceae

(F.N.Williams) Rabeler & W.L.Wagner comb. nov.

urn:lsid:ipni.org:names:77147459-1

Arenaria ischnophylla F.N.Williams, J. Linn. Soc., Bot. 38: 400. 1909.

##### Type.

CHINA. Xizang: Khamba Fort, 17 July 1903, F.E. Younghusband 107 (holotype, K, K000723883 [JSTOR image!]; E, E00317561 = photo of specimen at K!).

#### Eremogone juncea (M. Bieb.) Fenzl var. glabra

Taxon classificationPlantaeCaryophyllalesCaryophyllaceae

(Regel) Rabeler & W.L.Wagner comb. nov.

urn:lsid:ipni.org:names:77147461-1

Arenaria juncea var. glabra Regel, Bull. Soc. Imp. Naturalistes Moscou 35: 246. 1862.

##### Type.

CHINA: northern China, A.A. Tatarinoff (LE?).

#### Eremogone kumaonensis

Taxon classificationPlantaeCaryophyllalesCaryophyllaceae

(Maxim.) Rabeler & W.L.Wagner comb. nov.

urn:lsid:ipni.org:names:77147462-1

Arenaria kumaonensis Maxim., Fl. Tangut. 86. 1889.

##### Syntypes.

INDIA: Kumaon, Ralam valley, 12500 ft., R. Strachey & J.E. Winterbottom 3 (BM, BM000946338 [JSTOR image!]; K, K000742179 [JSTOR image!]; LE?); Ralam valley, 14000-16000 ft., 1884, J.F. Duthie 2757 (LE?); Tihri-Garhwal, J.F. Duthie s.n. (LE?).

#### Eremogone lancangensis

Taxon classificationPlantaeCaryophyllalesCaryophyllaceae

(L.H.Zhou) Rabeler & W.L.Wagner comb. nov.

urn:lsid:ipni.org:names:77147463-1

Arenaria lancangensis L.H.Zhou, Acta Phytotax. Sin. 18: 357. 1980.

##### Type.

CHINA. Yunnan: Weixi, 4000 m, T.T. Yü 8966 (holotype, PE).

#### Eremogone mukerjeeana

Taxon classificationPlantaeCaryophyllalesCaryophyllaceae

(Majumdar) Rabeler & W.L.Wagner comb. nov.

urn:lsid:ipni.org:names:77147464-1

Arenaria mukerjeeana (Majumdar) H.Hara, J. Jap. Bot. 51: 7. 1976, *Stellaria mukerjeeana* Majumdar, Blumea 16: 267. 1968.

##### Type.

NEPAL: Muktinath, 4250 m, T.B. Shrestha & Bista 2462A (holotype, CAL).

#### Eremogone potaninii

Taxon classificationPlantaeCaryophyllalesCaryophyllaceae

(Schischk.) Rabeler & W.L.Wagner comb. nov.

urn:lsid:ipni.org:names:77147465-1

Arenaria potaninii Schischk. in Komarov, Fl. URSS 6: 536. 1936, nom nov. for *Arenaria pentandra* Maxim., Bull. Acad. Imp. Sci. Saint-Pétersbourg 26: 429. 1880, non (J. Gay) Ardoino (1867), Turcz. (1834), Wallr. (1822), Dufour (1820).

##### Type.

Songaria, non procul a finibis Mongoliae, in montibus Kitschni-ne-tau, prope fortalitium Saissan, in rupibus, Potanin (LE?).

#### Eremogone pulvinata

Taxon classificationPlantaeCaryophyllalesCaryophyllaceae

(Edgew.) Rabeler & W.L.Wagner comb. nov.

urn:lsid:ipni.org:names:77147466-1

Arenaria pulvinata Edgew., Fl. Brit. India [J.D. Hooker] 1(2): 238. 1874.

##### Type.

INDIA. Sikkim: 14000-17000 ft. [Lana Kayna, 15000 ft., 24 July 1849 (on additional label on K sheet)], J.D. Hooker, Hooker & Thomson, Herb. Ind. Orient. 3 (Holotype, K, K000723992 [JSTOR image!]; isotypes, BM, BM000946339 [JSTOR image!], BM000946343 [JSTOR image!]).

#### Eremogone qinghaiensis

Taxon classificationPlantaeCaryophyllalesCaryophyllaceae

(Tsui & L.H.Zhou) Rabeler & W.L.Wagner comb. nov.

urn:lsid:ipni.org:names:77147467-1

Arenaria qinghaiensis Tsui & L.H.Zhou, Acta Phytotax. Sin. 18: 358. 1980.

##### Type.

CHINA. Qinghai: Dulan Xian, 4200 m, Qinghai-Gansu Exped. 1194 (holotype, WUG).

#### Eremogone roborowskii

Taxon classificationPlantaeCaryophyllalesCaryophyllaceae

(Maxim.) Rabeler & W.L.Wagner comb. nov.

urn:lsid:ipni.org:names:77147468-1

Arenaria roborowskii Maxim., Fl. Tangut. 87. 1889.

##### Type.

CHINA. Tibet: ad fl. Yang-tze, 20 July 1884, Przewalski? (LE?).

#### Eremogone shannanensis

Taxon classificationPlantaeCaryophyllalesCaryophyllaceae

(L.H.Zhou) Rabeler & W.L.Wagner comb. nov.

urn:lsid:ipni.org:names:77147469-1

Arenaria shannanensis L.H.Zhou in C.Y. Wu, Fl. Xizang. 1: 677. 1983.

##### Type.

CHINA. Xizang: Lunzhe, ca. 4300 m, B.Z. Guo et al. 22435 (holotype, NWBI).

#### Eremogone taibaishanensis

Taxon classificationPlantaeCaryophyllalesCaryophyllaceae

(L.H.Zhou) Rabeler & W.L.Wagner comb. nov.

urn:lsid:ipni.org:names:77147470-1

Arenaria taibaishanensis L.H.Zhou, Acta Phytotax. Sin. 18: 361. 1980.

##### Type.

CHINA. Shaanxi: Meixian, side of Dayehai, ca. 4000 m, Shaanxi Chin. Herb. Medic. Exped. 390 (holotype, WUG).

#### Eremogone zadoiensis

Taxon classificationPlantaeCaryophyllalesCaryophyllaceae

(L.H.Zhou) Rabeler & W.L.Wagner comb. nov.

urn:lsid:ipni.org:names:77147471-1

Arenaria zadoiensis L.H.Zhou, Acta Biol. Plateau Sin. 6: 26. 1987.

##### Type.

CHINA. Qinghai: Zado, ca. 4400 m, S.W. Liu 110 (holotype, WUG).

## Supplementary Material

XML Treatment for Eremogone

XML Treatment for Eremogone aksayqingensis

XML Treatment for Eremogone commagenae

XML Treatment for Eremogone depauperata

XML Treatment for Eremogone ferruginea

XML Treatment for Eremogone festucoides

XML Treatment for Eremogone festucoides (Benth.) Rabeler & W. L. Wagner var. imbricata

XML Treatment for Eremogone gerzensis

XML Treatment for Eremogone grueningiana

XML Treatment for Eremogone haitzeshanensis

XML Treatment for Eremogone ischnophylla

XML Treatment for Eremogone juncea (M. Bieb.) Fenzl var. glabra

XML Treatment for Eremogone kumaonensis

XML Treatment for Eremogone lancangensis

XML Treatment for Eremogone mukerjeeana

XML Treatment for Eremogone potaninii

XML Treatment for Eremogone pulvinata

XML Treatment for Eremogone qinghaiensis

XML Treatment for Eremogone roborowskii

XML Treatment for Eremogone shannanensis

XML Treatment for Eremogone taibaishanensis

XML Treatment for Eremogone zadoiensis
